# Targeting Soluble Epoxide Hydrolase and Cyclooxygenases Enhance Joint Pain Control, Stimulate Collagen Synthesis, and Protect Chondrocytes From Cytokine-Induced Apoptosis

**DOI:** 10.3389/fvets.2021.685824

**Published:** 2021-08-05

**Authors:** Laura Tucker, Troy N. Trumble, Donna Groschen, Erica Dobbs, Caroline F. Baldo, Erin Wendt-Hornickle, Alonso G. P. Guedes

**Affiliations:** ^1^Department of Veterinary Clinical Sciences, College of Veterinary Medicine, University of Minnesota, Saint Paul, MN, United States; ^2^Department of Veterinary Population Medicine, College of Veterinary Medicine, University of Minnesota, Saint Paul, MN, United States

**Keywords:** synovitis, arthritis, osteoarthritis, lameness, mobility, equine model

## Abstract

**Objective:** To determine the symptomatic and disease-modifying capabilities of sEH and COX inhibitors during joint inflammation.

**Methods:** Using a blinded, randomized, crossover experimental design, 6 adult healthy horses were injected with lipopolysaccharide (LPS; 3 μg) from *E. coli* in a radiocarpal joint and concurrently received the non-selective cyclooxygenase (COX) inhibitor phenylbutazone (2 mg/kg), the sEH inhibitor *t*-TUCB (1 mg/kg) or both (2 mg/kg phenylbutazone and 0.1, 0.3, and 1 mg/kg *t*-TUCB) intravenously. There were at least 30 days washout between treatments. Joint pain (assessed *via* inertial sensors and peak vertical forces), synovial fluid concentrations of prostanoids (PGE_2_, TxB_2_), cytokines (IL-1β, IL-6, TNF-α) and biomarkers of collagen synthesis (CPII) and degradation (C2C) were measured at pre-determined intervals over a 48-h period. The anti-apoptotic effect of COX and sEH inhibitors was determined *via* ELISA technique in primary equine chondrocytes incubated with TNF-α (10 ng/ml) for 24 h. Apoptosis was also determined in chondrocytes incubated with sEH-generated metabolites.

**Results:** Combined COX and sEH inhibition produced significantly better control of joint pain, prostanoid responses, and collagen synthesis-degradation balance compared to each compound separately. When administered separately, pain control was superior with COX *vs*. sEH inhibition. Cytokine responses were not different during COX and/or sEH inhibition. In cultured chondrocytes, sEH inhibition alone or combined with COX inhibition, but not COX inhibition alone had significant anti-apoptotic effects. However, sEH-generated metabolites caused concentration-dependent apoptosis.

**Conclusions:** Combined COX and sEH inhibition optimize pain control, attenuate loss of articular cartilage matrix during joint inflammation and cytokine-induced chondrocyte apoptosis.

## Introduction

Inflammation of the synovial lining (i.e., synovitis) and cartilage damage are prominent features of osteoarthritis (OA), strongly correlating with pain sensitization and disease severity. Synovitis can be present in all stages of OA, tends to worsen with radiographic severity and may contribute to the progression of cartilage damage (1–5). In equine and human OA, local and systemic release of cytokines such as tumor necrosis factor (TNF)-α and interleukin (IL)-6 are associated with pain (lameness) as well as greater loss of cartilage volume and joint space narrowing (6–9). As actively secreting cells, chondrocytes are especially susceptible to endoplasmic reticulum (ER) stress, an important mechanism leading to chondrocyte apoptosis (10–12) which also correlates with synovitis in OA (13).

**Figure 1:**
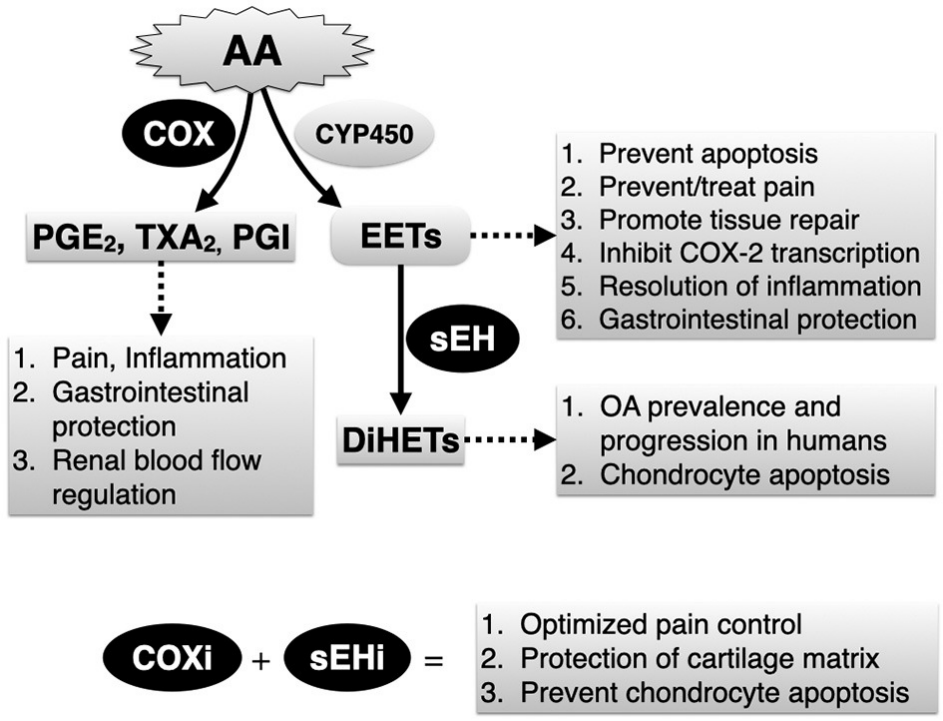
Simplified depiction of arachidonic acid (AA) metabolism *via* the cyclooxygenase (COX) and the epoxygenase (CYP450) pathways. Soluble epoxide hydrolase (sEH) is a critical yet relatively unexplored enzyme that breaks down endogenously produced and beneficial epoxyeicosatrienoic acids (EETs), generated by epoxygenases to their corresponding and potentially harmful dihydroxyeicosatrienoic acid (DiHETs). Main biology related to COX and sEH activities, and expected therapeutic outcomes related to combined COX inhibitors (COXi) and sEH inhibitors (sEHi) based on results of current study are listed.

Lameness and pain continue to be the primary complaint associated with OA in horses and humans. In horses and humans, cyclooxygenase (COX) inhibitors are first-line therapies to control OA pain yet this strategy is only partially effective and can cause serious adverse effects in both species (14, 15). In addition, COX inhibitors can induce ER stress and apoptosis (16), although evidence regarding their chondrotoxic or chondroprotective roles in humans remains conflicting (17). Recently, the arachidonic acid-derived metabolites 8,9-, 11,12-, and 14,15-dihydroxyeicosatrienoic acids (DiHETs), which are converted by soluble epoxide hydrolase (sEH) from the corresponding epoxyeicosatrienoic acid (EETs) regioisomers (Figure 1), were found to be significantly associated with the prevalence and progression of knee OA in older adults (18). This finding is important because a growing body of evidence suggests that, by preventing conversion of EETs to DiHETs, sEH inhibitors promote inflammatory resolution (19), antinociception (20–25), prevent ER stress and apoptosis (26–28), and support organ and tissue repair (29). In addition, in mouse models, sEH inhibitors lack addictive effects (30) and do not affect motor ability (25), result in similar or even greater antinociception compared to COX inhibitors (25) and morphine (22), display antinociceptive synergy with COX inhibitors (31) and can prevent COX inhibitor-induced intestinal ulceration (32). Thus, sEH inhibition alone or combined with COX inhibition could represent a significant development in the management of OA by conferring both symptom- and disease-modifying effects.

Our laboratory has previously demonstrated that sEH is involved in joint pain and physical disability using equine models of joint inflammation (33) and naturally-occurring models of chronic pain (laminitis) (34, 35) that, similar to human (36) and equine (37) OA, is characterized by inflammatory and neuropathic changes (38). We have also determined that the sEH inhibitor *t*-TUCB (*trans*-4-{4-[3-(4-Trifluoro-methoxy-phenyl)-ureido]-cyclohexyloxy}-benzoic acid) and several others developed for the human enzyme are equally potent against equine sEH (35) and that *t*-TUCB has a good plasma pharmacokinetic profile in horses, achieving therapeutically relevant concentrations within the equine joint (33). Recently, pharmacologic inhibition of sEH ameliorated hyperalgesia, edema, and expression of pro-inflammatory cytokines in joint tissues of a mouse model of rheumatoid arthritis (39). Another study in laboratory Beagles with naturally-occurring OA showed a statistically significant, albeit modest, improvement in subjective measures of pain and mobility (40). Further, incubation of cultured canine chondrocytes with EETs attenuated IL-1β-induced IL-6 and TNF-α secretion and reduced cytotoxicity (40). However, no study has examined the symptomatic (pain) and disease modifying effects of sEH and COX inhibition in the context of painful joint diseases.

The goals of the present study were to assess the symptomatic and disease-modifying capabilities of sEH and COX inhibitors on articular tissues. We hypothesized that combined COX and sEH inhibition would attenuate joint pain on ambulation and the breakdown of articular cartilage matrix associated with synovitis, and would prevent cytokine-induced chondrocyte apoptosis to a significantly greater degree than inhibiting COX or sEH separately.

## Materials and Methods

### Experimental Animals

Six adult horses (5 castrated males, 1 sexually intact female) aged 8.5 ± 3 years (range 5–13 years old) and weighing 462 ± 50 kg (range 397–536 kg) were used. Horses were considered healthy and free of radiocarpal joint disease on the basis of complete veterinary work-up that included general physical and orthopedic examinations, complete blood cell counts and plasma chemistry profile. Horses were housed as a group on paddocks for the duration of the study with water available *ad libitum* and grass hay fed once/daily. The University of Minnesota Institutional Animal Care and Use committee reviewed and approved the study protocol.

### Test Compounds

The non-selective COX inhibitor, phenylbutazone, was obtained from Vedco Inc. (EQUI-PHAR, Phenylbutazone injection 20%; Saint Joseph, MO, USA) or from Sigma-Aldrich, Inc. (Saint Louis, MO, USA), and the 8,9-, 11,12-, and 14,15-DiHETs were obtained from Cayman Chemical (Ann Arbor, MI, USA). The sEH inhibitor, *t*-TUCB, was kindly provided by Dr. Bruce Hammock (University of California-Davis), synthesized and characterized according to established methodology (41–44). Equine recombinant TNF-α was purchased from R&D Systems (Minneapolis, MN, USA).

### Equine Radiocarpal Synovitis Model and Drug Treatments

Horses were sedated (xylazine, 0.2–0.5 mg/kg IV; AnaSed, Akorn Inc., IL, USA) and synovitis was induced by injecting 2 ml of a freshly prepared solution of lipopolysaccharide (LPS) from *E. coli* O55:B5 (1.5 μg/ml in 0.9% NaCl; total 3 μg; catalog number L5418, Sigma-Aldrich, St. Louis, MO, USA) into one randomly assigned radiocarpal joint for the first injection, with subsequent injections alternating between joints, as in a previous study from our laboratory (33). There were at least 30 days washout between subsequent LPS injections (and treatments). Following a randomized crossover experimental design in which each horse served as its own control, treatments consisted of the non-selective COX inhibitor phenylbutazone (PBZ, 2 mg/kg; 0.01 ml/kg of a 200 mg/ml commercial solution), the sEH inhibitor *t*-TUCB (1 mg/kg) or a combination of both drugs (2 mg/kg PBZ and 1, 0.3, or 0.1 mg/kg *t*-TUCB). We intentionally chose a relatively moderate dose of phenylbutazone to avoid fully blocking pain or prostanoid production, reasoning that this would allow interactions with the varying doses of the sEH inhibitor to be revealed. *t*-TUCB was dissolved in dimethyl sulfoxide (100% DMSO, Sigma-Aldrich) to final concentrations of 100, 30, and 10 mg/ml and filter-sterilized with 0.2 μm pore size sterilizing-grade membranes prior to administration (0.01 ml/kg). Drugs were administered slowly (30–45 s) as a single intravenous injection using separate jugular vein catheters, at the same time that the joints were injected with LPS. In the phenylbutazone-only treatment, horses also received the vehicle diluent of *t*-TUCB (DMSO, 0.01 ml/kg) to control for possible DMSO anti-inflammatory effects (45, 46) Horses in the *t*-TUCB-only treatment group also received an intravenous injection of 0.9% saline (0.01 ml/kg). Treatment responses were determined by assessing lameness (pain on ambulation) in a straight-line trot on a hard, flat surface. Kinematic parameters were assessed by calculating the vector sum of the head height difference relative to the stride cycle using an inertial sensor system (Lameness Locator, Equinosis LLC, Columbia, MO, USA) (47, 48) and the peak vertical force (PVF) using an in-ground force platform system (AMTI, Watertown, MA, USA). Prior to (i.e., baseline) and at 2, 4, 8, 24, 32, and 48 h after LPS/drug administration horses were trotted to achieve a minimum of 25 total strides for kinematic analysis, and 5 acceptable trials per set of ipsilateral fore and hind limbs on the force plate within 2.8–3.3 m/s and an acceleration <10%. If the horse was unable to bear weight on the LPS injected limb for a trial period, then the maximum decrease in vector sum was inputted (−137), as per the manufacturer's recommendation, and the PVF was recorded as zero for that time.

### Synovial Fluid Biomarkers

Baseline synovial fluid was collected from the radiocarpal joint immediately prior to injection with LPS. Subsequent arthrocenteses were performed 8, 24, and 48 h after injection with LPS/treatments. All samples were collected without dilution, centrifuged, aliquoted, and stored at −80°C until further analyses. To determine the effect of COX and sEH inhibition on articular cartilage matrix, prostanoid production, and pro-inflammatory biomarkers during synovitis, we assayed (ELISA) all synovial fluid samples for type II collagen synthesis (CPII) and degradation (C2C) biomarkers (IBEX Technologies, Quebec, CAN), prostaglandin (PG) E_2_, thromboxane (Tx) B_2_ (Enzo Life Sciences, Farmingdale, NY, USA), interleukin (IL)-β, IL-6, and tumor necrosis factor (TNF)-α (equine specific assays from Genorise Scientific, Inc., Glen Mills, PA, USA). Use of equine synovial fluid with type II collagen biomarkers have been previously validated (49), as have the cytokine biomarkers by the manufacturer; these assays were measured without digestion. Type II collagen synthesis-degradation balance was assessed by calculating CPII to C2C ratios. Measurements were performed as per the manufacturer's recommendations, at necessary dilutions, in duplicate or triplicate. Mean intra-assay coefficients of variations (CV) for the biomarkers were: CPII 3.1%, C2C 3.9%, PGE_2_ 10.7%, TxB_2_ 4.3%, IL-1β 5.2%, IL-6 2.4%, and TNFα 2.1%, and inter-assay CVs were: CPII 9.0%, C2C 12.4%, PGE_2_ 4%, TxB_2_ 5.8%, IL-1β 12.9%, IL-6 7.0%, and TNFα 8.0%.

### Chondrocyte Cultures

Primary equine articular chondrocytes of second passage (American Research Products, Inc., Waltham, MA, USA) were cultured in high-glucose Dulbecco's Modified Eagle Medium (Gibco Laboratories, Gaithersburg, MD, USA) supplemented with 10% heat-inactivated fetal calf serum and 1% penicillin/streptomycin (Sigma-Aldrich) in an atmosphere with 5% CO_2_ at 37°C until reaching ~80–90% confluency and then passaged to 12-well culture dishes at a density of 10,000 cells/ml for 24 h. Next, the chondrocytes were changed to serum-free medium and incubated with TNF-α (10 ng/ml) along with the COX inhibitor PBZ, the sEH inhibitor *t*-TUCB or both at several concentrations for 24 h. The inhibitors were used at their approximate half-maximal inhibitory concentration or IC_50_ (*t*-TUCB 4 nM, PBZ 4 μM), 80% inhibitory concentration or IC_80_ (*t*-TUCB 40 nM, PBZ 40 μM) and 10-fold > IC_80_ (*t*-TUCB 400 nM, PBZ 400 μM) for the equine enzymes (35, 50). In additional experiments, chondrocytes were incubated for 24 h in serum-free medium with several concentrations (0, 1, 10 ng/ml) of the sEH-generated 8,9-, 11,12-, and 14,15-DiHETs. Controls were treated with 0.9% saline (vehicle diluent for TNF-α) and ethanol (vehicle diluent for PBZ, *t*-TUCB and DiHETs; 0.0001% final concentration in culture medium). At the end of the 24-h incubation period, cells were harvested and apoptosis was determined in duplicates using ELISA technique as per the manufacturer's instructions (Cell Death Detection ELISA^PLUS^, Roche GmbH, Mannheim, Germany).

### Statistics

Data were analyzed using GraphPad Prism (GraphPad Software, La Jolla, CA). Areas under the curves (AUC) were calculated using the trapezoidal method for some of the variables, as indicated in the Results section. Data from the *in vivo* studies were analyzed using linear mixed-effects model whereby treatment and time were included as fixed factors and horse as random factor if normally distributed, or the Friedman test if not. Normality was determined primarily by visual inspection of QQ plots and the Shapiro-Wilk normality test. Chondrocyte apoptosis data were considered normally distributed by visual inspection of QQ plots and the Shapiro-Wilk normality test, and were analyzed by One- or Two-way ANOVA. In all cases, the two-stage step-up method of Benjamini, Krieger, and Yekutieli was used as multiple comparison test while correcting for multiple comparisons by controlling the False Discovery Rate. The Geisser-Greenhouse correction was applied for data showing different scatter. Since there were missing data for TxB_2_, IL-1β, IL-6, and TNF-α, the number of analyzed data points is indicated in the text. Significance level was set at *p* < 0.05. Data are presented as mean±SEM.

## Results

### Combined sEH and COX Inhibition Resulted in Better Control of Joint Pain During Synovitis Than Inhibiting Each Enzyme Separately

**Figure 2:**
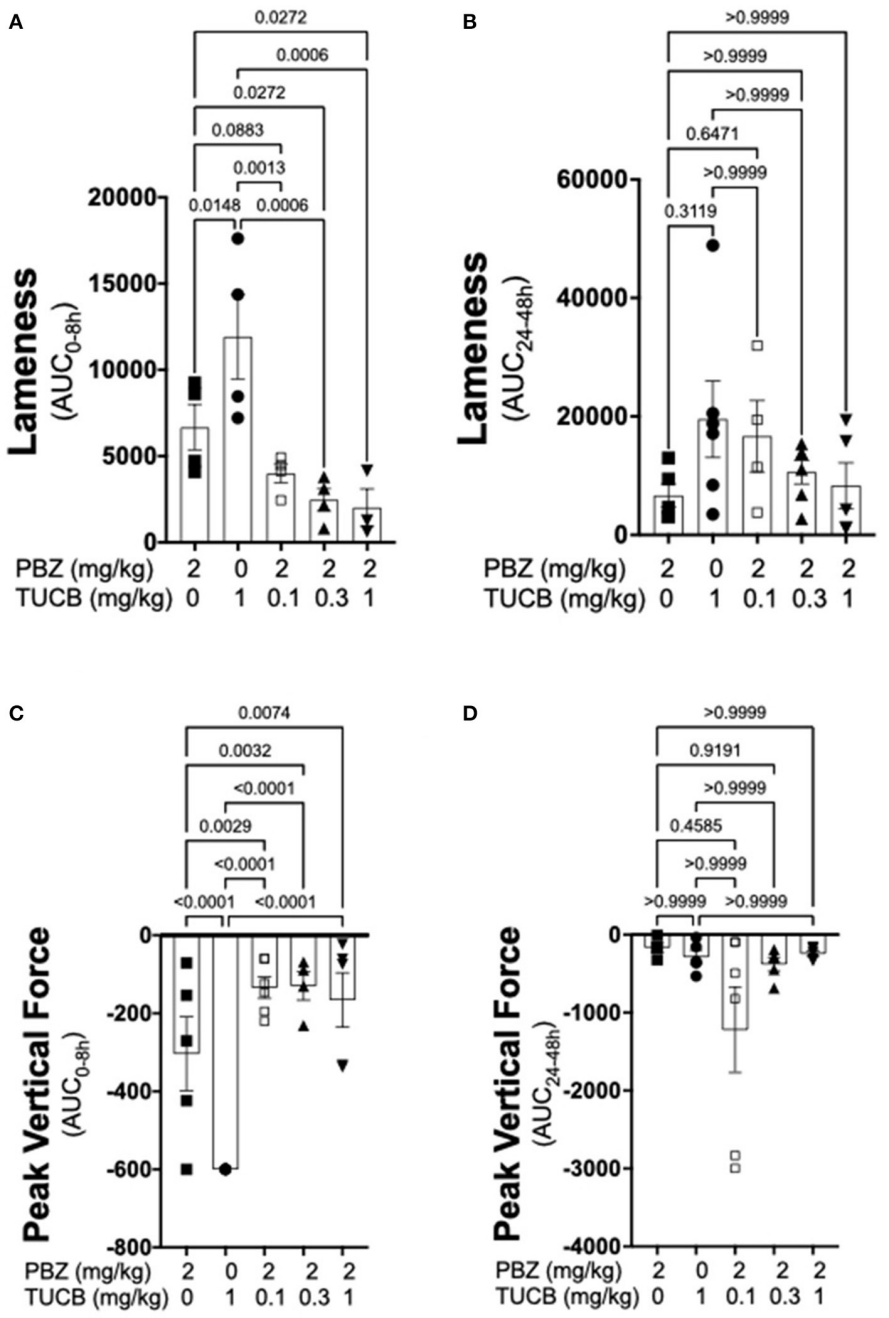
Combined sEH and COX inhibition resulted in better control of joint pain in the early phase of synovitis than inhibiting each enzyme separately. Pain was estimated as a change in lameness that was objectively assessed *via* inertial sensors **(A,B)** as well as forces applied to an in-ground force platform **(C,D)** during the early, 0–8 h **(A,C)**, and late 24–48 h **(B,D)** phases of synovitis induced by intra-articular injection of lipopolysaccharide (LPS; 3 μg) into the radiocarpal joint in horses (*n* = 6). Horses were treated intravenously with the sEH inhibitor *trans*-4-{4-[3-(4-Trifluoro-methoxy-phenyl)-ureido]-cyclohexyloxy}-benzoic acid (TUCB), the non-selective COX inhibitor phenylbutazone (PBZ) or both, at the indicated doses marked below the graphs, at the same time as the intra-articular LPS injection. Data are shown as individual values (symbols) and mean ± SEM (columns) of the 8 h **(A,C)** and 24 h **(B,D)** aggregate area under the curve (AUC) corrected for baseline (% change). *P*-values are shown above each comparison (Mixed-effects model, **(A,C)**; Friedman test, **(B,D)**; Corrected for multiple comparisons by controlling the False Discovery Rate).

Results of kinematic and kinetic parameters are shown in Figure 2. We analyzed these data in two separate periods (0–8 and 24–48 h) because joint pain/lameness induced by LPS is transient, peaking at 8–12 and resolving thereafter even without treatment (33) and the duration of action of the selected dose of the COX inhibitor phenylbutazone to control joint pain in horses is ~8–12 h (51). In the first 8 h following induction of synovitis, pain on ambulation assessed objectively with inertial sensors was significantly less with combined COX and sEH inhibition than when each enzyme was inhibited separately. Further, sEH inhibition alone was significantly less efficacious than COX inhibition alone. From 24–48 h after synovitis induction, there were no significant differences among treatments. Simultaneous assessment of peak vertical forces using an in-ground force platform system yielded similar results. Thus, based on internally consistent results obtained with two separate, unbiased and objective measures of joint pain during ambulation, our findings suggest that COX inhibition provides superior control of synovitis-associated joint pain than sEH inhibition, and that concurrently inhibiting both enzymes result in significantly better control of joint pain than inhibiting each enzyme separately.

### Effect of COX and sEH Inhibition on Inflammatory Biomarkers

**Figure 3:**
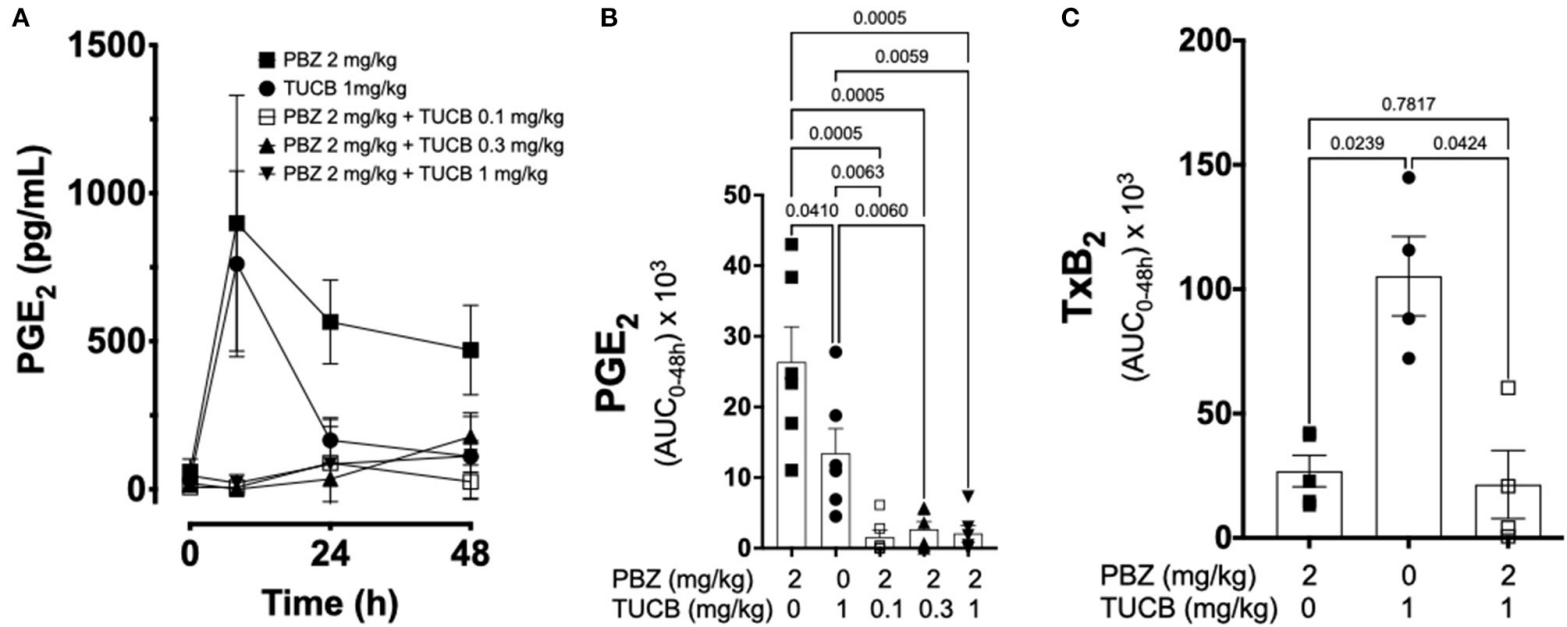
Effect of soluble epoxide hydrolase (sEH) and cyclooxygenases (COX) inhibition on prostanoid response in joint fluid during synovitis. Forty-eight-hour time course **(A)** and aggregate area under curve (AUC) **(B)** for synovial fluid concentrations of prostaglandin (PG) E_2_, and thromboxane A_2_ AUC **(C)** in an equine synovitis model induced by intra-articular injection of lipopolysaccharide (LPS; 3 μg) into the radiocarpal joint (*n* = 6 horses) as measured using ELISA techniques. Horses were treated intravenously with the sEH inhibitor *trans*-4-{4-[3-(4-Trifluoro-methoxy-phenyl)-ureido]-cyclohexyloxy}-benzoic acid (TUCB), the non-selective COX inhibitor phenylbutazone (PBZ) or both, at the indicated doses marked below the graphs, at the same time as the intra-articular LPS injection. For graph **(A)** data are shown as mean ± SEM. For graphs **(B,C)** data are shown as individual values (symbols) and mean ± SEM (columns), and *p*-values are shown above each respective comparison [Friedman test **(B)**; Mixed-effects model **(C)**; Corrected for multiple comparisons by controlling the False Discovery Rate].

**Figure 4:**
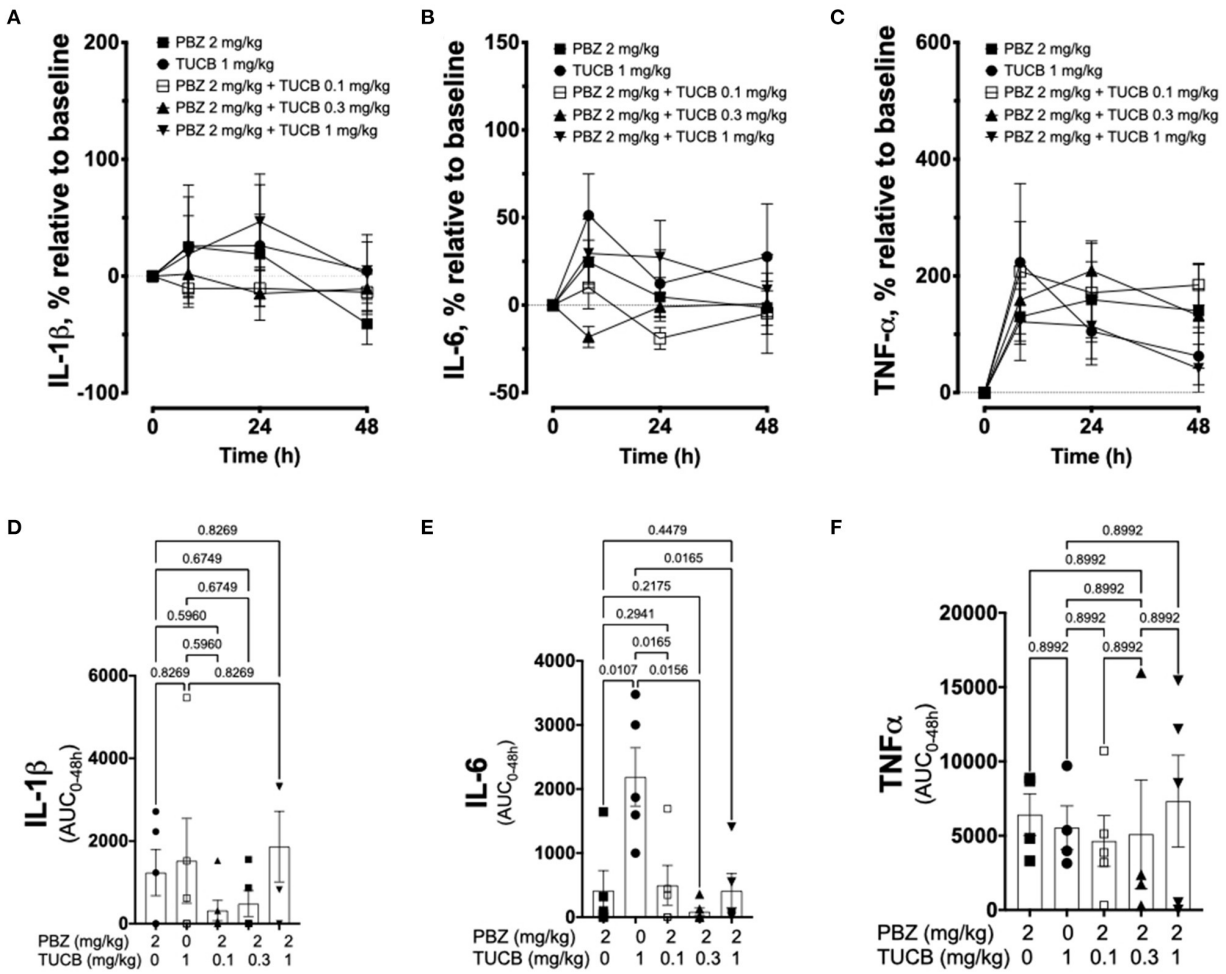
Cytokine response associated with synovitis during inhibition of soluble epoxide hydrolase (sEH) and cyclooxygenases (COX). Forty-eight-hour time course (top row) and aggregate area under curve (AUC, bottom row) for synovial fluid concentrations of interleukin (IL)-1β **(A,D)**, IL-6 **(B,E)**, and tumor necrosis factor (TNF)-α **(C,F)** in an equine synovitis model induced by intra-articular injection of lipopolysaccharide (LPS; 3 μg) into the radiocarpal joint (*n* = 6 horses) as measured using ELISA techniques. Horses were treated intravenously with the sEH inhibitor *trans*-4-{4-[3-(4-Trifluoro-methoxy-phenyl)-ureido]-cyclohexyloxy}-benzoic acid (TUCB), the non-selective COX inhibitor phenylbutazone (PBZ) or both, at the indicated doses marked above (top row) or below (bottom row) the graphs, at the same time as the intra-articular LPS injection. Top row: Data are shown as mean ± SEM. Bottom row: Data are shown as individual values (symbols) and mean ± SEM (columns), and *p*-values are shown above each respective comparison (Mixed-effects model corrected for multiple comparisons by controlling the False Discovery Rate).

We quantified several biomarkers of inflammation to compare the extent that COX and sEH inhibition modified the joint responses to synovitis. We did not divide these data into separate periods for statistical analyzes because joint inflammatory response in the LPS-induced synovitis model peaks at 12–24 h and lasts for at least 48 h (33). As shown in Figure 3, synovial fluid concentrations of PGE_2_ were significantly lower during treatment with combined inhibition of COX and sEH compared to COX or sEH inhibition individually. All doses of sEH significantly potentiated the inhibition of PGE_2_ production by the COX inhibitor. We also determined the synovial fluid concentrations of TxB_2_ during treatment with the highest dose of the sEH inhibitor (1 mg/kg *t*-TUCB) alone or when combined with the COX inhibitor (*n* = 4/6 horses). Results showed that TxB_2_ levels were not significantly different between COX inhibition alone and the combined COX and sEH inhibition, but was significantly higher than either of these during sEH inhibition alone. Thus, sEH inhibition added a significant inhibitory effect on COX-2-generated PGE_2_ but not on COX-1-generated TxB_2_. We also assessed cytokine responses within the joint (Figure 4) with emphasis on IL-1β (*n* = 4/6 horses), IL-6 (*n* = 4–6/6 horses), and TNF-α (*n* = 5–6/6 horses), given their demonstrated association with joint pain and cartilage loss in OA (2, 7, 9, 52–54). Unexpectedly, there were no significant differences between COX, sEH or combined COX and sEH inhibition on cytokine release in synovial fluid, except for IL-6, which was significantly higher during sEH inhibition alone compared to COX inhibition alone and the combined COX and sEH inhibition. Notably, TNF-α levels increased 100–200% above baseline with all treatments. Taken together, these findings suggest that combined COX and sEH inhibition produced better control of PGE_2_ synthesis in response to synovitis than inhibiting each enzyme separately, whereas there was no added benefit of sEH inhibition upon cytokine release.

### Combined COX and sEH Inhibition Provides Superior Protection of the Articular Cartilage Matrix Than Inhibiting COX Alone

**Figure 5:**
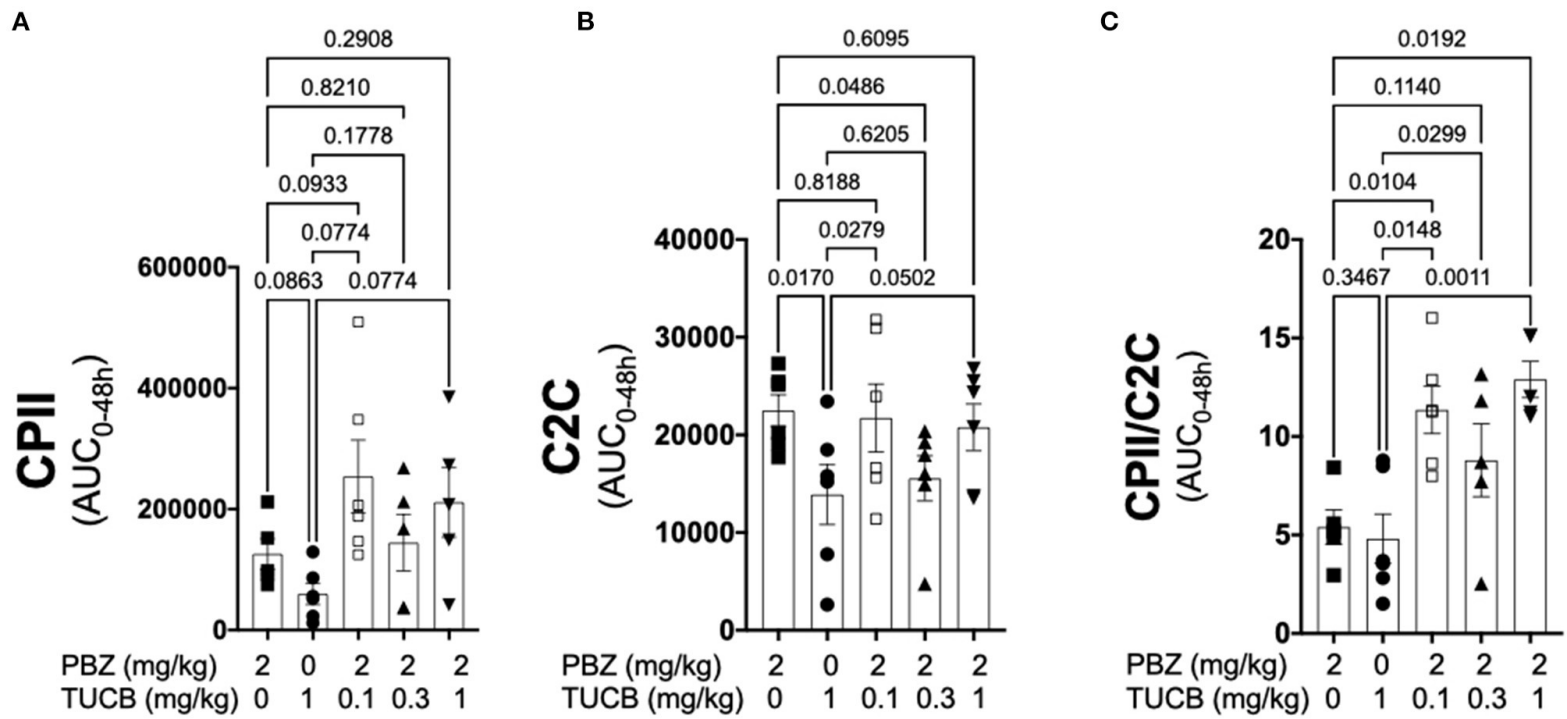
Combined inhibition of soluble epoxide hydrolase (sEH) and cyclooxygenases (COX) during joint inflammation favored collagen synthesis over degradation. Synovial fluid concentrations of the biomarker of type II collagen synthesis CPII **(A)**, type II collagen degradation C2C **(B)** and the synthesis-degradation ratio **(C)** calculated based on 48-h aggregate concentrations (AUC) in synovial fluid from an equine synovitis model induced by intra-articular injection of lipopolysaccharide (LPS; 3 μg) into the radiocarpal joint (*n* = 6 horses) as measured using ELISA techniques. Horses were treated intravenously with the sEH inhibitor *trans*-4-{4-[3-(4-Trifluoro-methoxy-phenyl)-ureido]-cyclohexyloxy}-benzoic acid (TUCB), the non-selective COX inhibitor phenylbutazone (PBZ) or both, at the indicated doses marked below the graphs, at the same time as the intra-articular LPS injection. Data are shown as individual values (symbols) and mean ± SEM (columns), and *p*-values are shown above each respective comparison (Mixed-effects model corrected for multiple comparisons by controlling the False Discovery Rate).

Since synovitis and joint inflammation alters the normal collagen synthesis-degradation coupling and contribute to loss of articular cartilage matrix and OA development (1, 4, 13), we sought to determine the effect of COX and sEH inhibition on the collagen-degradation relationship during synovitis. As shown in Figure 5, CPII concentrations were not significantly different during inhibition of COX, sEH, or both whereas C2C concentrations were significantly lower during sEH inhibition compared to COX inhibition and the lowest dose of combined COX and sEH inhibition. The resulting synthesis-degradation balance favored synthesis by ~10-fold during combined COX and sEH inhibition compared to only ~5-fold when COX or sEH were inhibited separately. The synthesis-degradation ratio was higher during combined COX and sEH inhibition compared to either COX or sEH inhibition alone.

### Inhibition of sEH Prevents TNF-α-Mediated Chondrocyte Apoptosis and sEH-Generated Metabolites Cause Chondrocyte Apoptosis

**Figure 6:**
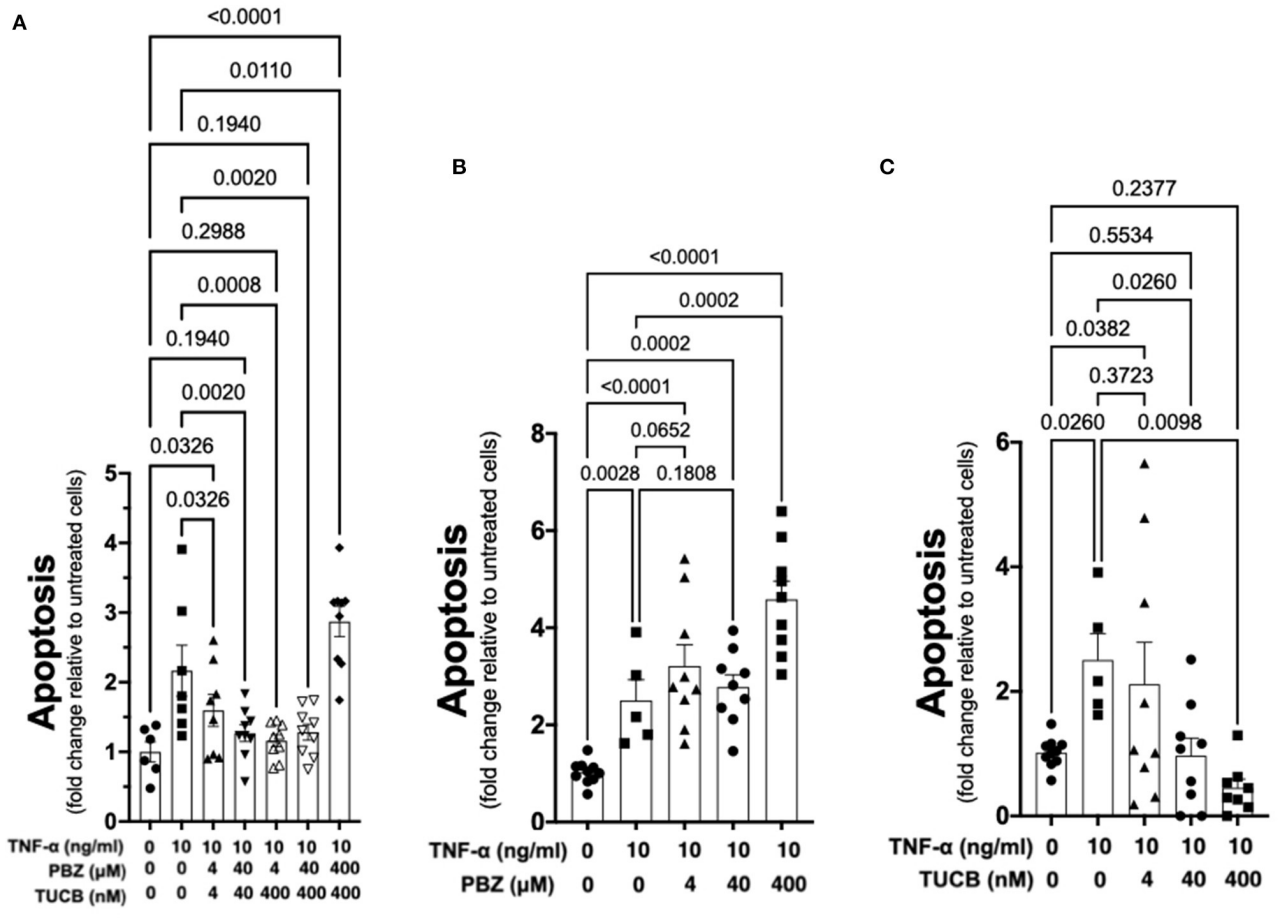
Soluble epoxide hydrolase (sEH) inhibition prevents cytokine-induced chondrocyte apoptosis. Primary equine articular chondrocytes were incubated with TNF-α (10 ng/ml) for 24 h along with several concentrations of the cyclooxygenase (COX) inhibitor phenylbutazone (PBZ) combined with the sEH inhibitor *trans*-4-{4-[3-(4-Trifluoro- methoxy-phenyl)-ureido]-cyclohexyloxy}-benzoic acid (TUCB) **(A)**, with PBZ alone **(B)** or with TUCB alone **(C)**. PBZ and TUCB were used at their approximate half-maximal inhibitory concentration or IC_50_ (4), IC_80_ (40), and 10-fold > IC_80_ (400) for the equine enzymes. Negative controls were treated with 0.9% saline (vehicle diluent for TNF-α) or ethanol (vehicle diluent for PBZ and TUCB; 0.0001% final concentration in culture medium). Apoptosis was determined using ELISA technique as per the manufacturer's instructions. Concentrations are listed below each graph. Data are shown as individual values (symbols) and mean ± SEM (columns), and *p*-values are shown above each comparison (One-way ANOVA corrected for multiple comparisons by controlling the False Discovery Rate).

**Figure 7:**
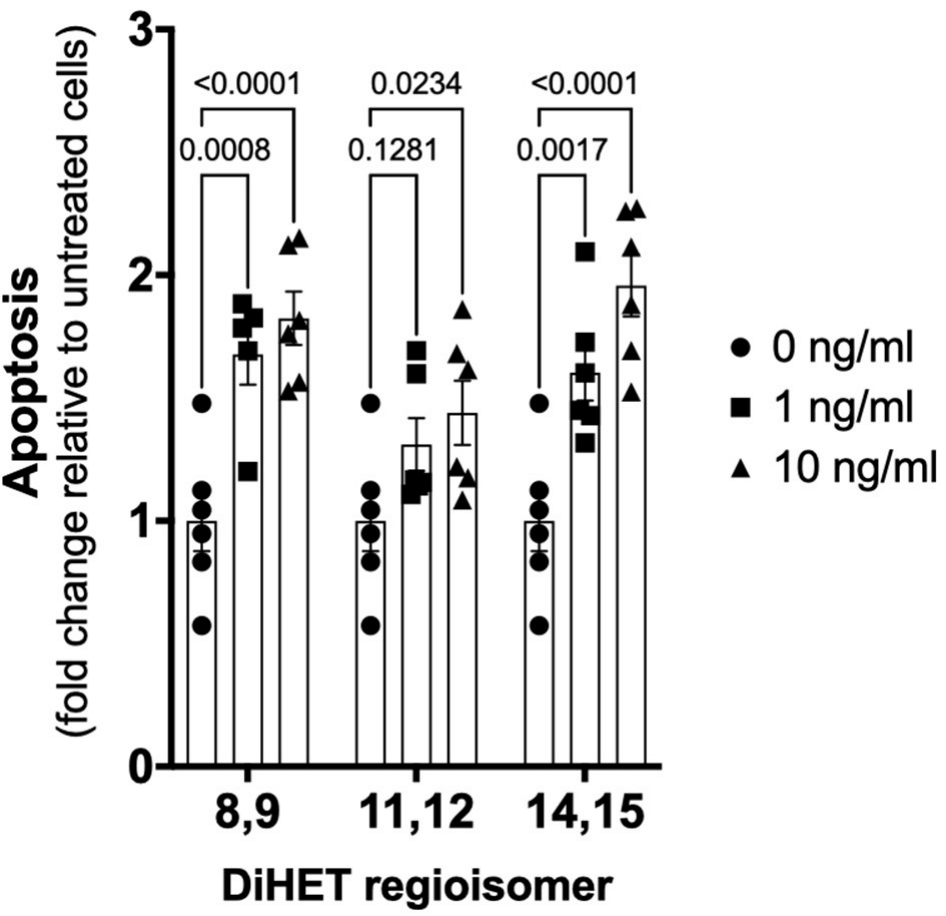
Soluble epoxide hydrolase (sEH)-generated metabolites from arachidonic acid cause chondrocyte apoptosis. Primary equine articular chondrocytes were incubated with the sEH-generated dihydroxy-eicosatrienoic acid (DiHETs) 8,9, 11,12, and 14,15 regioisomers for 24 h at the indicated concentrations. Controls (0 ng/ml) were treated with vehicle diluent (ethanol; 0.0001% final concentration in culture medium). Apoptosis was determined using ELISA technique as per the manufacturer's instructions. Data are shown as individual values (symbols) and mean ± SEM (columns), and *p*-values are shown above each comparison (Two-way ANOVA corrected for multiple comparisons by controlling the False Discovery Rate).

As shown in Figure 6, TNF-α-induced apoptosis of primary equine articular chondrocytes was prevented by combined COX and sEH inhibition. This finding did not hold true, however, when the two inhibitors were used at 10-fold their respective IC_80_ in which case the inhibitor combination appeared to potentiate apoptosis. Chondrocytes were also treated separately with COX and sEH inhibitors to determine their individual anti-apoptotic contribution, revealing that COX inhibition with phenylbutazone did not prevent and even appeared to potentiate TNF-α-induced apoptosis at the highest concentration tested. On the contrary, sEH inhibition with *t*-TUCB resulted in a concentration-dependent protection of the chondrocytes from TNF-α-induced apoptosis. Lastly, as shown in Figure 7, chondrocytes developed significant apoptosis when incubated with sEH-generated DiHETs but in particular the 8,9- and 14,15-DiHETs.

## Discussion

The current study provides the first experimental evidence of the pain and disease-modifying capabilities of sEH and COX inhibitors during inflammation of an articular joint. Known analgesic mechanisms of sEH inhibitors include indirect transcriptional inhibition of COX-2 expression, activation of neurosteroid transcription in the central nervous system (22) and opioid-dependent signaling (55). sEH inhibitors have no direct pharmacologic effect on COX-2 activity (22). As such, combination therapy with COX and sEH inhibitors result in multimodal analgesia that should be more effective than single modality therapy. Consistent with this notion, our results demonstrated that combined COX and sEH inhibition attenuated joint pain and the breakdown of articular cartilage matrix associated with synovitis more effectively than inhibiting COX or sEH separately. However, protection against cytokine-induced chondrocyte apoptosis was not greater with combined COX and sEH inhibition compared to sEH inhibition alone.

We employed objective and unbiased measures of lameness and ground-reaction forces as indirect readouts for synovitis-associated joint pain. Inhibition of both COX and sEH, rather than blocking each enzyme separately, was required for optimum control of synovitis-induced joint pain, and that sEH inhibition alone is less effective in improving joint pain than COX inhibition alone. The significantly lower efficacy of sEH inhibition compared to COX inhibition contrasts with earlier findings in rodent models of inflammatory pain (25), whereas the superior results obtained with combined COX and sEH inhibition is consistent with previous reports in mice (31) and rats (21). The increased production of antinociceptive EETs due to the shift in arachidonic acid carbon flow toward the P450 and sEH pathways caused by COX inhibition (31), which are prevented from degradation by sEH inhibition, likely explain the enhanced analgesia when the activities of both enzymes are blocked. In the absence of sEH inhibition, sEH rapidly converts EETs to DiHETs, inactivating their antinociceptive activities (21, 22, 56). Thus, our findings suggest that sEH inhibition alone does not provide superior relief of synovitis-associated joint pain compared to COX inhibition alone, but pain relief is greatest when both enzymes are inhibited concurrently.

In our study, combined COX and sEH inhibition was significantly more effective in blocking COX-2-generated PGE_2_ than COX inhibition alone. While PGE_2_ has biologically relevant activities such as increased vascular permeability that facilitate edema formation and leukocyte infiltration that could be important in the setting of joint infections, these are likely not desirable in OA. Furthermore, there is significant correlation between OA and PGE_2_-responsive signaling pathways in human articular cartilage (57). These results are consistent with the multimodal effect of the inhibitor combination on COX-2 activity, including direct pharmacologic inhibition by phenylbutazone and transcriptional inhibition by *t*-TUCB (22). Since phenylbutazone is a non-selective COX inhibitor, we also measured COX-1-generated TxB_2_ to determine if the COX and sEH inhibitors interacted to affect COX-1 activity. The sEH inhibitor had no significant inhibition of COX-1 as indicated by (i) similar TxB_2_ levels between combined COX and sEH inhibition and COX inhibition alone, and (ii) significantly higher TxB_2_ during sEH inhibition alone. Taken together, these results demonstrate that sEH inhibition added a significant inhibitory effect on COX-2, but not on COX-1, during synovitis, which should mitigate the activation of PGE_2_-responsive signaling pathways in chondroncytes (57).

To better understand the effects COX and sEH inhibition on other pro-inflammatory biomarkers relevant to OA, we also determined the synovial fluid concentrations of several cytokines and only found a significant difference with IL-6 during sEH inhibition alone. This result contrasts with prior studies in mice showing that sEH inhibition significantly attenuated serum TNF-α protein during systemic endotoxemia (19) and joint tissue TNF-α and IL-1β messenger RNA levels in whole joint tissues in a model of rheumatoid arthritis (39). It is difficult to reconcile these disparities given the different methodologies and species studied. However, our results are likely more relevant to humans because the equine synovial joint shares greater similarity with human joints than that of mice, both histologically and in terms of biomarkers (58). In adddition, we determined cytokine protein levels in synovial fluid, which is an important compartment due to its direct contact with the chondrocytes that are lost during OA. An intriguing result was the significant increase in IL-6 concentration during sEH inhibition alone. The IL-6 biology is complex and may involve both pro- and anti-inflammatory activities, the degree of which depend on the magnitude of IL-6-*trans*-signaling *via* its soluble receptor (sIL-6R) vs. “classical” signaling *via* its membrane bound receptor (mIL-6R) (59). Investigating the reasons for the increased IL-6 concentrations during sEH inhibition was beyond the scope of our study, but it may represent a response to increased levels of gp130, the transducer molecule for soluble sIL-6R and mIL-6R. Increased gp130 binds and inactivates the IL-6/soluble IL-6R complex as well as insulate its signaling molecules, driving the concentration of IL-6 to elevate sufficiently for signaling to occur (59). A decrease in IL-6 signaling protects against cartilage matrix damage and OA development (54). Further, the increased IL-6 levels within the joint may explain the lower antinociceptive efficacy of sEH inhibition alone compared to the other treatments because IL-6 is involved in peripheral nociceptor sensitization and pathological pain (60).

We also sought to determine whether or not COX and/or sEH inhibition could protect the articular cartilage matrix during synovitis because (i) type II collagen degradation by matrix metalloproteinases (MMP) correlates with symptomatic radiographic and pre-radiographic OA (61), (ii) cytokines that increased during synovitis in our study (e.g., IL-1β, TNF-α) and toll-like receptor ligands (e.g., LPS) are known to induce MMP expression (62), and (iii) sEH activity has been linked to OA prevalence and progression (18). Inhibition of sEH alone significantly suppressed type II collagen degradation (C2C) compared to inhibition of COX alone, although the resulting synthesis-degradation balance was similar to COX inhibition because it also slightly attenuated collagen synthesis (CPII). Since sEH inhibitors are known to block activation of NF-κB (63), a transcription factor involved in MMP expression (62), the observed significant decrease in collagen degradation suggests that sEH inhibitors might decrease MMP activity. Most importantly, combined COX and sEH inhibition significantly favored synthesis over degradation compared to inhibiting COX or sEH alone. Since the CPII-C2C ratio is indicative of progression/non-progression of OA (64), a therapeutic approach with combined COX and sEH inhibition has the potential to mitigate degradation of articular cartilage matrix and slow or prevent OA progression.

The medical treatment of OA focuses primarily on alleviating pain symptoms, and no currently available therapy also targets the joint pathology and progressive cartilage damage. In our study, targeting sEH in addition to COX during synovitis and joint inflammation resulted in significantly better pain control, lower synovial fluid concentration of PGE_2_ and improved collagen synthesis-degradation balance. These are important findings because PGE_2_ is pro-nociceptive, activating PGE_2_-responsive signaling pathways in chondrocytes that sensitizes them to apoptosis (57, 65, 66), and destruction of the articular cartilage matrix also predisposes chondrocytes to apoptosis in response to cytokines such as TNF-α (67). Since TNF-α is strongly associated with cartilage loss in OA (7, 52) and was poorly controlled with COX and sEH inhibition in our synovitis model, we tested the effects of COX and sEH inhibition on TNF-α-induced apoptosis of primary equine articular chondrocytes. The combined inhibition of COX and sEH had a significant anti-apoptotic effect, which was confered by the sEH inhibitor since it displayed concentration-dependent anti-apoptotic effect whereas the COX inhibitor either did not prevent or even increased chondrocyte apoptosis. Further, the sEH-generated DiHETs caused significant chondrocyte apoptosis, consistent with their previously reported association with knee OA in older adults (18). An increased DiHET production during COX inhibition due to the higher arachidonic acid carbon flow toward the P450 and sEH pathways (31) might explain apoptosis by COX inhibitors (16). Taken together with the findings of Valdes and colleagues linking sEH metabolites and OA (18), we suggest that blocking the conversion of EETs to DiHETs with sEH inhibitors will prevent chondrocyte death in naturally-occuring OA, mitigating cartilage damage. Such therapy would represent a significant advance in the medical care of OA.

Our study has several possible limitations worth considering. The majority of our horses were castrated males and only one was a sexually intact female, thus the study was not balanced by sex or gonadal status. While there has been no study examining gonadal status or sex predilection for OA in horses, the condition tends to be more prevalent in older women than in men and is attributed to changes in sex hormones associated with menopause (68). The reproductive cycle of female horses can be influenced by photoperiod (69), a notable difference from that of human females. Testosterone levels in our male castrated horses were presumably lower than if they were gonadally intact. Higher testosterone levels were associated with less pain in severe knee OA in men and women and less disability in women (70). Our sample size was relatively small and the LPS-induced synovitis model used produces a transient but moderate to severe inflammation and joint pain (33). This is unlikely to be the type/degree of synovitis encountered in many naturally-occurring OA cases, and thus our results might represent the more extreme forms of OA. On the other hand, the LPS-induced synovitis model is relevant because systemic and local concentrations of LPS have been associated with macrophage activation in the knee joint capsule and synovium as well as with the severity of structural abnormalities and symptoms of knee OA in humans (71). Finally, the treatment sequence was not modeled in the statistical analyzes to control for a possible increased permeability of the microvascular endothelium in the more frequently injected joints. However, this is unlikely to be an important factor based on our previous experience using a similar experimental design in this synovitis model (33), which ensures that the same joint is injected no >60 days apart allow the synovial fluid protein levels and leukocyte counts to return to pre-injection values even after multiple injections.

In summary, our study provides multiple lines of evidence suggesting that combined COX and sEH inhibition provides an effective mode of therapy in this large animal model. Our findings indicate that this therapeutic strategy would enhance symptomatic pain control and attenuate loss of articular cartilage matrix and apoptotic death of chondrocytes, ultimately resulting in reduced cartilage damage and disease progression.

## Data Availability Statement

The original contributions presented in the study are included in the article/supplementary material, further inquiries can be directed to the corresponding author.

## Ethics Statement

The animal study was reviewed and approved by University of Minnesota Institutional Animal Care and Use Committee.

## Author Contributions

AG and TT carried out the analysis and interpretation of the data. LT, DG, ED, CB, EW-H, and AG collected and assembled the data. TT and AG drafted the manuscript. LT, TT, DG, ED, CB, and EW-H revised the article for important intellectual content. TT and AG obtained funding for conceived and designed the experiments. All authors provided final approval of the article prior to submission.

## Conflict of Interest

The authors declare that the research was conducted in the absence of any commercial or financial relationships that could be construed as a potential conflict of interest.

## Publisher's Note

All claims expressed in this article are solely those of the authors and do not necessarily represent those of their affiliated organizations, or those of the publisher, the editors and the reviewers. Any product that may be evaluated in this article, or claim that may be made by its manufacturer, is not guaranteed or endorsed by the publisher.
